# Assessing tolerability with the Functional Assessment of Cancer Therapy item GP5: psychometric evidence from LIBRETTO-531, a phase 3 trial of selpercatinib in medullary thyroid cancer

**DOI:** 10.1186/s41687-024-00823-8

**Published:** 2024-12-19

**Authors:** Antoine Regnault, Laurine Bunod, Angely Loubert, Marcia S. Brose, Lisa M. Hess, Patricia Maeda, Yan Lin, Rebecca M. Speck, Adrienne M. Gilligan, Nalin Payakachat

**Affiliations:** 1 Modus Outcomes, a THREAD Company, Lyon, France; 2 Sidney Kimmel Medical College, Thomas Jefferson University, Philadelphia, PA USA; 3 Eli Lilly and Company, Lilly Corporate Center DC 1730, Indianapolis, IN 46285 USA

**Keywords:** Medullary thyroid cancer, Patient-reported tolerability, Psychometric analysis, FACT item GP5

## Abstract

**Background:**

This psychometric analysis generated evidence to support the use of the Functional Assessment of Cancer Therapy item GP5 (GP5) as a measure of tolerability and confirms the appropriateness of categorizing “high side-effect burden” using a rating of 3 or 4 (score ranges 0–4) in patients with advanced/metastatic *RET-*mutant medullary thyroid cancer (MTC).

**Methodology:**

Blinded, pooled interim data from the safety population (*n*=290) enrolled in the phase 3 LIBRETTO-531 trial (NCT04211337) were used. Intraclass correlation coefficients (ICC) were calculated for test-retest reliability using data from cycles 1-2 post-baseline. Construct validity was evaluated by examining the correlations of GP5 ratings with (a) symptomatic adverse events (AEs; measured by the PRO-CTCAE), and (b) functioning scores of EORTC QLQ-C30. The ability to detect change over time was examined by Cochrane-Mantel-Haenszel tests for GP5 ratings and PRO-CTCAE. The relationship of “high side-effect burden” categories with QLQ-C30 functioning scores was examined.

**Results:**

ICCs for the GP5 ratings after cycle 1 ranged between 0.80 and 0.85, indicating good reliability. Correlations between GP5 and PRO-CTCAE items ranged from 0.18 to 0.62 and ranged from -0.37 to -0.50 for QLQ-C30 functioning scores, consistent with study assumptions. Post-baseline GP5 ratings showed significant associations with PRO-CTCAE scores (*p*<0.001). Participants with GP5 ratings of 3 or 4 had worse physical function than those with GP5 ratings of 0 to 2 (*p*<0.0001).

**Conclusions:**

This analysis generated evidence supportive of the psychometric properties of the GP5 as a fit-for-purpose measure to assess treatment tolerability in patients with advanced/metastatic MTC. The definition of “high side-effect burden” was associated with the clinical feature of tolerability.

**Supplementary Information:**

The online version contains supplementary material available at 10.1186/s41687-024-00823-8.

## Key summary points

This psychometric analysis provides evidence to support the usefulness of the categorization of “high side-effect burden” using GP5 scores of 3 and 4.

The GP5 has good reliability and construct validity for assessing patient-reported tolerability.

This analysis supports the use of the GP5 to assess patient-reported tolerability in patients with advanced or metastatic medullary thyroid cancer.

**Supplementary Information:**

The online version contains supplementary material available at 10.1186/s41687-024-00823-8.

## Introduction

Trialist, regulatory, medical, and patient communities are increasingly focused on capturing the direct patient perspective on the side effects of treatment and the impact of these side effects on their life and overall well-being in clinical trials [1]. Symptomatic adverse events (AE) and overall side effect impact are now recognized as core patient reported outcomes (PROs) in cancer clinical trials by the FDA [2].

The concept of tolerability is inherently patient-centered; it can be usefully distinguished from the clinician-centered concept of treatment safety [3]. Multiple stakeholders, including regulators (both US and EU), researchers, patients, and sponsors, define tolerability as “the degree to which symptomatic and non-symptomatic adverse events associated with the product’s administration affect the ability or desire of the patient to adhere to the dose or intensity of therapy” [1, 3]. Two complementary approaches have been proposed for the measurement of patient-reported tolerability [1]. The first approach involves collecting the experience of patients with each side effect relevant to a specific treatment or class. Item banks such as the PRO version of Common Terminology Criteria for Adverse Events (PRO-CTCAE) library [4] or the European Organization for Research and Treatment of Cancer (EORTC) Item Library are validated resources for researchers to select items that assess potential AEs tailored to treatments in their clinical trials [5]. A second approach is to use a single-item measure to assess the overall impact of treatment side effects. The best candidates include two items extracted from common measurement systems: the Functional Assessment of Cancer Therapy General item 5 (GP5; “I am bothered by side effects of treatment”) [6], and the item Q168 of the EORTC Item Library (“To what extent have you been troubled with side-effects from your treatment?”) [6].

LIBRETTO-531 is a global, multi-center, randomized (2:1), open-label, Phase 3 study comparing selpercatinib to physician’s choice of cabozantinib or vandetanib in patients with progressive, advanced, tyrosine kinase inhibitor (TKI)-naïve, rearranged during transfection (*RET)*-mutation positive medullary thyroid cancer (MTC) [6]. MTC accounts for approximately 1 to 2% of thyroid cancer cases in the United States [7]. In LIBRETTO-531, comparative tolerability was an alpha-controlled secondary PRO endpoint. Comparative tolerability was assessed using GP5 post-baseline scores of 3 or 4, which was defined as “high side-effect burden,” and by comparing the proportion of time on treatment with high side-effect burden between the two treatment arms [8].

Although psychometric evidence on the GP5 item has been generated in a variety of settings [9–11], additional evidence is needed to support its use as a fit-for-purpose measure of patient-reported tolerability in patients with MTC. Therefore, this analysis of the GP5 was conducted to demonstrate the psychometric components of the measure as being appropriate for assessing patient-reported tolerability and to evaluate appropriateness of the categorization of “high side-effect burden” based on a response of 3 or 4 in the setting of advanced or metastatic MTC.

## Methods

### Data

Blinded, pooled data was drawn from an interim data cut from the LIBRETTO-531 phase 3 trial (NCT04211337). Details of the methods and results of this clinical trial are described elsewhere [12, 13]. This analysis used the safety population (*n*=290), which included all randomized patients who received at least 1 dose (including a partial dose) of study treatment.

### Patient reported outcome measures

PRO data used in this psychometric analysis of the GP5 included: (a) the EORTC Quality of Life Questionnaire – core 30 items (EORTC QLQ-C30), (b) the PRO-CTCAE, and (c) EQ-5D-5L.

The GP5, a single-item measure, uses a 5-point Likert scale: 0 (not at all); 1 (a little bit); 2 (somewhat); 3 (quite a bit); or 4 (very much), with a 7-day recall period [6].

The EORTC QLQ-C30 is a 30-item validated measure assessing functions, symptoms, and health-related quality of life (HRQoL) in patients with cancer [14, 15]. It includes five functional scales (physical, role, cognitive, emotional, and social), three symptom scales (fatigue, pain, and nausea and vomiting), a global health status/Quality of Life (GHS/QoL) scale, and six single items assessing additional symptoms commonly reported by patients with cancer. Higher functional scores represent better functioning ability, higher GHS/QoL scores represent better QoL, and higher symptom scores represent more severe symptoms.

The PRO-CTCAE was developed to evaluate self-reported symptomatic toxicity in patients with cancer [16]. LIBRETTO-531 included twenty PRO-CTCAE items covering thirteen symptomatic AEs anticipated from both treatment arms: dry mouth, mouth or throat sores, tasting food or drink, decreased appetite, nausea, vomiting, constipation, diarrhea, rash, acne, hand-foot syndrome, headache, and fatigue.

The EQ-5D-5L comprises a descriptive system and a visual analogue scale (VAS) to measure health status [17]. The descriptive system assesses five dimensions of health status: mobility, self-care, usual activities, pain/discomfort, and anxiety/depression.

These PRO measures were self-administered by patients aged 18 or older who were literate in an available translation of each measure. All PRO measures were administered electronically using provisioned handheld device at baseline (Cycle 1 Day 1) and then weekly (GP5 and PRO-CTCAE) or once every 28 days (EORTC QLQ-C30 and EQ-5D-5L) during the on-treatment study period.

### Psychometric analysis

Reliability or reproducibility was investigated within a test-retest framework, based on the agreement between patient scores at two timepoints, to estimate the ability of the GP5 to produce a stable rating among patients with a stable condition. In the context of assessing reliability of the GP5, the stable condition was determined using patients who reported no more than one point change on PRO-CTCAE ratings at the same assessment timepoints as the GP5. Intraclass correlation coefficients (ICCs) were calculated between successive GP5 assessments of cycles 1 and 2. Specifically, test-retest reliability was estimated between Cycle 1 Day 1 and Day 8; Cycle 1 Day 8 and Day 15; Cycle 1 Day 15 and Day 22; Cycle 1 Day 22 and Cycle 2 Day 1; Cycle 2 Day 1 and Day 8; Cycle 2 Day 8 and Day 15; and Cycle 2 Day 15 and Day 22. The main test-retest reliability was focused on the ICCs in Cycle 2 given the anticipated variability in Cycle 1 and the need to allow for patients to adjust to treatment AEs and reporting of symptomatic AEs.

ICCs were calculated using a two-way mixed-effects, absolute agreement, single measurement approach [18]. Kappa coefficients, with Cicchetti Allison correction, were also calculated to determine the agreement between these consecutive assessments to account for the ordinal scale of the GP5 [19]. ICCs ≥0.8 were considered adequate [20] and Kappa coefficient estimates greater than 0.6 were considered substantial agreement [21, 22].

Construct validity of the GP5, including convergent validity and known-group validity, was assessed by examining its association with other parameters, using a priori hypotheses guided by the definition of tolerability provided by Friends of Cancer Research [1]. It was hypothesized that more severe symptomatic AEs would be associated with higher overall quality of life burden and that poorer functioning and GHS/QoL would be associated with higher overall side-effect burden. The concepts assessed by the EORTC QLQ-C30 and EQ-5D-5L are not directly related to side-effect bother; they were therefore expected to be only mildly to moderately associated with the GP5 (typical hypothesized correlation coefficient between 0.2 and 0.4). Among the concepts assessed by the EORTC QLQ-C30, physical functioning was expected to be the most associated with side-effect bother. Convergent validity was assessed by Spearman rank correlation between the GP5 rating and both the PRO-CTCAE and QLQ-C30 (i.e., functioning scores and GHS/QoL scores) from Cycles 1 to 5, with the emphasis on the correlations at Cycle 3 (i.e., allowing stability of symptomatic AE reporting). Known-group validity of the GP5 was evaluated by describing the distribution of GP5 ratings at the four assessments before and the first assessment after (a) treatment discontinuation in patients who discontinued treatment due to AE or personal decision and (b) hospitalization among those who were hospitalized. Benchmark (i.e., a reference group) distributions for purposes of comparison and to aid interpretation were created by pooling GP5 ratings over the first five cycles of treatment from all patients, excluding those who discontinued treatment or had a dose modification or were hospitalized.

The ability of the GP5 to detect change over time was examined by describing the change in GP5 ratings in groups of patients based on their change in PRO-CTCAE responses from baseline. An indicator of a worsening symptomatic AE was created for each participant at each time when the PRO-CTCAE and GP5 were simultaneously collected between baseline to Cycle 5 Day 1. If the participant reported a worsening of categories in any PRO-CTCAE item compared to baseline, they were classified as having a worsened symptomatic AE; if not, they were classified as stable. GP5 ratings at each weekly assessment from baseline to Cycle 5 were cross tabulated with baseline GP5 ratings in participants with worsened symptomatic AEs and in participants with stable symptomatic AEs, separately. Cochrane-Mantel-Haenszel tests were then performed to examine associations between post-baseline GP5 ratings and worsening of PRO-CTCAEs, adjusted for baseline GP5 ratings.

### Categorization of “high side-effect burden”

A series of statistical analyses were designed to inform the categorization of “high side-effect burden” based on GP5 ratings. The primary categorization specified that a GP5 rating of 3 or 4 indicates high side-effect burden. Two exploratory categorizations were tested to provide points of comparison for the primary categorization: the first considered that only a GP5 response of 4 indicated high side-effect burden and the second considered GP5 ratings of 2, 3 or 4 indicated high side-effect burden.

Descriptive analyses included the number and duration of the longest period per patient with high side-effect burden and a description of the EORTC QLQ-C30 functioning and GHS/QoL scores by “high side-effect burden” or not by the primary and each exploratory categorization. Comparison of QLQ-C30 scores between all patients with “high side-effect burden” and those without was conducted using analysis of variance (ANOVA) for continuous variables.

All statistical analyses were performed using SAS V9.4 (SAS Institute; Cary, NC, USA).

## Results

In the safety population (*n*=290), the average age was 54.2 (SD=14.1) years, and the majority were male (62.8%) and of White race (69.4%) (Supplementary Table 1).

### Reliability of the GP5

The ICCs estimated for the GP5 after the first cycle in a group of stable patients were consistently ≥0.8, indicating adequate reliability (Table 1). The agreement between consecutive GP5 ratings over the same period, as indicated by the Kappa coefficients, was also adequate.

**Table 1:** Test-retest reliability of the GP5

Timepoints for comparison of GP5 ratings	Patients with change in PRO-CTCAE items of no more than 1 category between the two considered timepoints		
	*N*	ICC	Kappa coefficient
Cycle 1 Day 1 and Cycle 1 Day 8	49	0.35	0.33
Cycle 1 Day 8 and Cycle 1 Day 15	108	0.67	0.56
Cycle 1 Day 15 and Cycle 1 Day 22	116	0.60	0.54
Cycle 1 Day 22 and Cycle 2 Day 1	122	0.74	0.61
Cycle 2 Day 1 and Cycle 2 Day 8	138	0.80	0.72
Cycle 2 Day 8 and Cycle 2 Day 15	138	0.81	0.68
Cycle 2 Day 15 and Cycle 2 Day 22	143	0.85	0.75

*ICC* intraclass correlation coefficient

### Construct validity of the GP5

This pattern of correlations was as expected. Spearman correlation coefficients between the GP5 and PRO-CTCAE items ranged from 0.18 (vomiting) to 0.62 (fatigue) across all assessments, suggesting low to moderate correlations (Table 2) [23].

**Table 2:** Correlation coefficients between GP5 and PRO-CTCAE items across all assessments

PRO-CTCAE item	Correlation coefficient^a^	
	All available GP5 assessments Number of assessments=13,619	
Acne or Pimples – Severity	0.31	
Constipation – Severity	0.25	
Decreased Appetite - Severity	0.45	
Decreased Appetite - Interfere	0.47	
Diarrhea – Frequency	0.30	
Dry Mouth – Severity	0.49	
Fatigue – Severity	0.62	
Fatigue – Interfere	0.61	
Hand and Foot Syndrome – Severity	0.45	
Headache – Frequency	0.38	
Headache – Severity	0.37	
Headache – Interfere	0.42	
Mouth or Throat Sores – Severity	0.40	
Mouth or Throat Sores - Interfere	0.43	
Nausea – Frequency	0.38	
Nausea – Severity	0.38	
Rash – Boolean	0.26	
Tasting Food or Drink – Severity	0.53	
Vomiting – Frequency	0.18	
Vomiting – Severity	0.18	

^a^Spearman rank-order correlation coefficients

Spearman correlation coefficients ranged from -0.37 to -0.50 for the relationship between the GP5 and the QLQ-C30 in the pooled cycles (Supplementary Table 2).

**Fig. 1:**
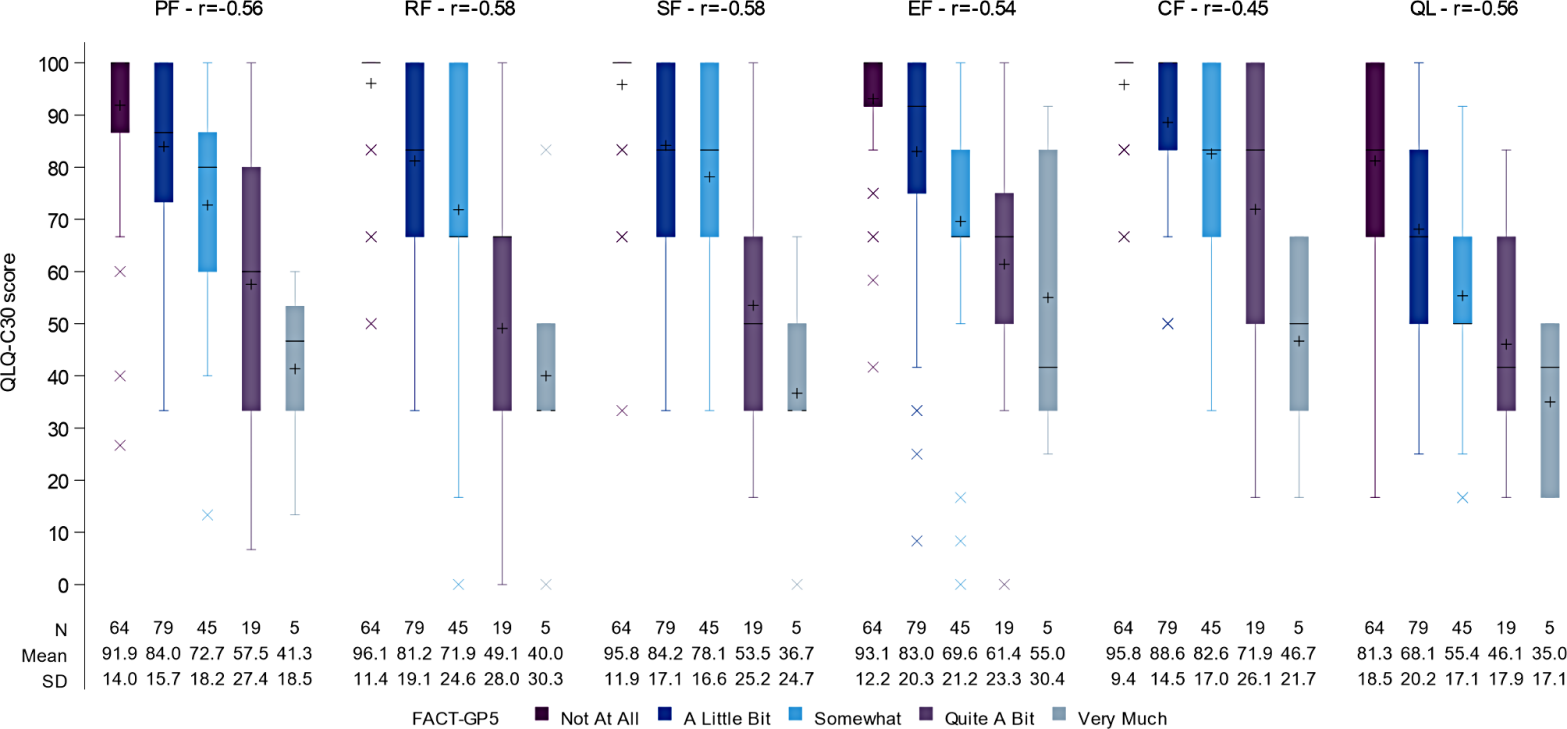
Distribution of QLQ-C30 functioning and quality of life/global health scores according to GP5 at cycle 3

Figure 1 displays the distribution of QLQ-C30 functioning scores according to the GP5 at cycle 3. Overall, the distributions of the QLQ-C30 functioning and GHS/QoL scores across GP5 ratings were as expected. The mean±SD of the QLQ-C30 Physical Functioning (PF) score was 91.9±14.0 in patients who reported on GP5 being “not at all” bothered by side effects (*n*=64), 84.0±15.7 in patients who reported being “a little bit” bothered (*n*=79), 72.7±18.3 in patients who reported being “somewhat” bothered (*n*=45), 57.5±27.4 in patients who reported being “quite a bit” bothered (*n*=19), and 41.3±18.5 in patients that reported being “very much” bothered (*n*=5).

**Fig. 2:**
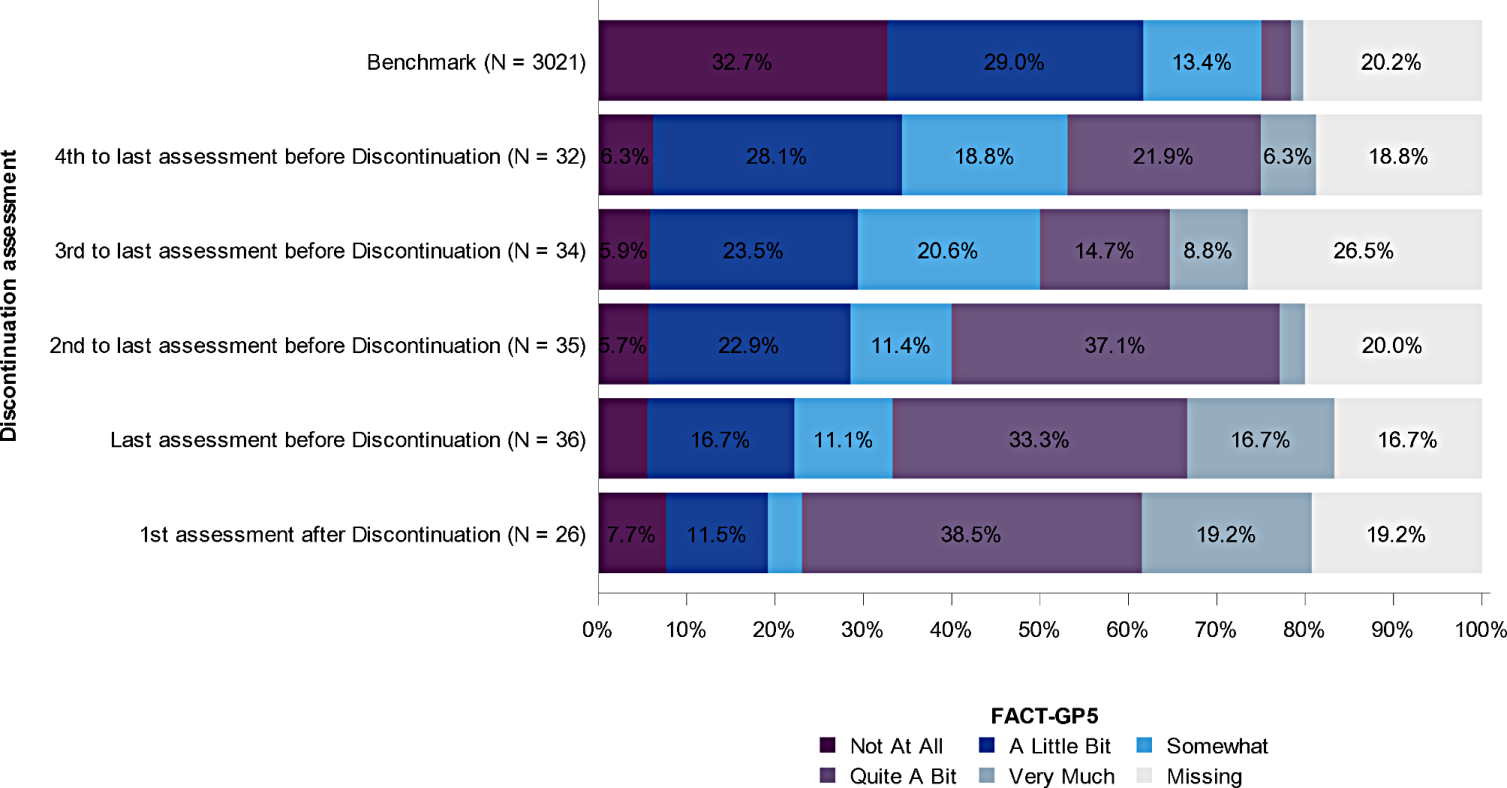
Distribution of the GP5 at the 4 assessments before treatment discontinuation and first assessment after treatment discontinuation in patients who discontinued treatment due to AE or personal decision, in relation the to the benchmark group

Figure 2 displays the distribution of GP5 ratings among patients who discontinued treatment due to AE (*n*=30) or personal decision (*n*=6) at the four assessments before treatment discontinuation and the first assessment after treatment discontinuation. The level of side-effect burden was higher at the assessment closer to treatment discontinuation, with 28.1% (*n*=9) of patients reporting “quite a bit” or “very much” bother at the 4^th^ to last assessment before discontinuation, 23.5% (*n*=8) at the 3^rd^ to last assessment before discontinuation, 40.0% (*n*=14) at the 2^nd^ to last assessment before discontinuation, 50.0% (*n*=18) at the last assessment before discontinuation, and 57.7% (*n*=15) at the 1^st^ assessment after discontinuation. The proportion of patients experiencing higher levels of side effect burden among those who discontinued treatment are much greater in magnitude than that of the benchmark sample, of whom only 4.7% experienced “quite a bit” or “very much” burden. No association was observed between GP5 rating and treatment adherence (data not shown).

Higher side-effect burden was reported in the last assessment before and the first assessment after hospitalization (Supplementary Fig. 1) compared to those who were not hospitalized (benchmark group).

### Ability of the GP5 to detect change

There were statistically significant (*p* < 0.001) relationships between the GP5 and the PRO-CTCAE, controlling for the GP5 rating at baseline. More than half of patients with worsened AEs from baseline that reported “not at all” bother at baseline (54%) reported “somewhat”, “quite a bit”, or “very much” bother at Cycle 5 Day 1, versus 7.5% in those that had stable AEs from baseline (Supplementary Figs. 2 and 3).

### Exploration of the categorization of “high side-effect burden” based on GP5

Of the 290 patients, 129 (44.5%) experienced high side-effect burden at least one point in the study according to the primary categorization of a GP5 rating of 3 or 4 (Table 3). 19% (*n*=55) of patients experienced high side-effect burden according to the exploratory categorization #1 of a GP5 rating of 4, and 67.6% percent of patients (*n*=196) experienced high side-effect burden using the exploratory categorization #2 of a GP5 rating of 2, 3, or 4 (Table 3).

**Table 3:** Longest period of high side-effect burden among patients who reported high side-effect burden for at least one assessment during the treatment period

Variable	High side-effect burden definition		
	GP5 of 3 or 4	GP5 of 4	GP5 of 2, 3 or 4
	*n*=129	*n*=55	*n*=196
Longest high side-effect burden duration (weeks)			
*n*	129	55	196
Mean (SD)	8.45 (14.94)	3.60 (4.68)	16.13 (21.78)
Median	3.00	1.00	6.00
Q1, Q3	2.00, 6.00	1.00, 3.00	2.00, 18.50
Min, Max	1.00, 82.00	1.00, 23.00	1.00, 99.00

*SD* standard deviation

**Fig. 3:**
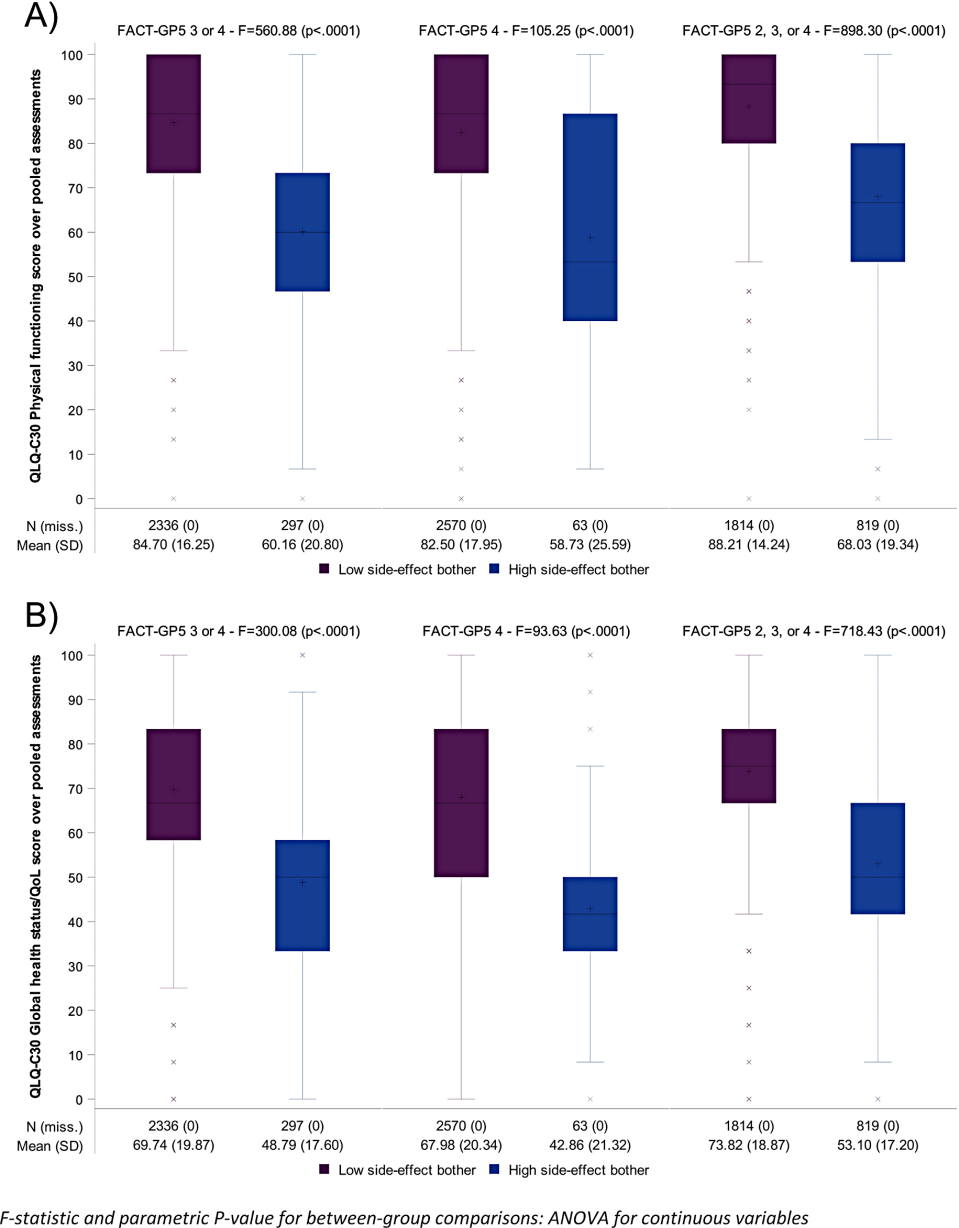
QLQ-C30 Physical functioning (Panel A) and Quality of Life/Global health status (Panel B) score according to high side-effect burden. F-statistic and parametric *P*-value for between-group comparisons: ANOVA for continuous variables

When considering all available assessments (a total of 2,633 assessments for which both the GP5 and the QLQ-C30 PF scores were available), 297 (11.3%) assessments were categorized as high side-effect burden with the primary categorization (Fig. 3). Sixty-three (2.4%) assessments were categorized as high side-effect burden when limited to a GP5 rating of 4, and 819 (31.1%) of assessments were categorized as high side-effect burden when defined as a GP5 rating of 2, 3, or 4 (Fig. 3).

Table 3 presents the longest period of high side-effect burden during the treatment period in patients who reported high side-effect burden for at least one assessment. According to the primary categorization (GP5 of 3 or 4), the median duration of the longest period of high side-effect burden was 3.0 weeks. The longest period was 1.0 week for GP5 exploratory categorization of 4 and was 6.0 weeks for GP5 exploratory categorization of 2, 3, or 4.

Figure 3 displays the distribution of the QLQ-C30 PF (Panel A) and QLQ-C30 GHS/QoL (Panel B) scores according to high side-effect burden categorizations. Patients having high side-effect burden per the primary categorization (GP5 of 3 or 4) experienced poorer functioning and QOL. Based on the primary categorization, the mean±SD of the QLQ-C30 PF score was 60.2±20.8 at the time of assessment when the participant was categorized as experiencing high side-effect burden and was 84.7±16.2 for those with GP5 less than 3 (*p*<0.0001). (Based on the primary categorization of a GP5 rating of 3 or 4, the mean±SD of the QLQ-C30 GHS/QoL score was 48.8±17.6 at the time of assessment when the participant was categorized as experiencing high side-effect burden and was 69.7±19.9 for those who did not report side-effect burden under this categorization (*p*<0.0001).

## Discussion

This study showed that the GP5 has acceptable measurement properties (i.e., reliability, validity, and ability to detect change) in accordance with current FDA guidance on patient-focused drug development [24]. Specifically, this psychometric evidence, along with the categorization of “high side-effect burden” based on a GP5 response of 3 (“Quite a bit”) or 4 (“Very much”), support the use of the GP5 to assess patient-reported tolerability in clinical trials of patients with advanced or metastatic MTC and in the context-of-use for assessing the comparative tolerability endpoint in LIBRETTO-531.

The psychometric findings are consistent with existing literature [9–11]. The reliability coefficient estimates obtained in the test-retest analysis were greater than those previously reported (0.37-0.61) [11]. Lower ICC and Kappa coefficient estimates observed in the first cycle in this study likely reflected the higher variability in the experience of AEs over the first weeks of treatment. However, the reliability coefficient estimates for the GP5 should be interpreted with caution since it is a single-item measure, and such measures are known to have poorer reliability than multiple item scales.

The correlation levels with other PRO measures assessing functioning and HRQoL that were obtained in this study are similar to those previously reported (between 0.3 and 0.5) [9, 11]. The association of the GP5 rating with the patient-reported severity of individual symptomatic AEs evaluated using the PRO-CTCAE items in this study was heterogenous, as observed previously, reflecting the variable burden of the various AEs experienced by patients. This represents a consistent body of evidence of the good psychometric properties of the GP5 across various cancers, including MTC.

One of the key strengths of these results compared to previously published data is the collection of more granular data (i.e., GP5 weekly assessments were available). Therefore, the test-retest reliability estimates were likely more accurate as they were evaluated from two consecutive weeks, where the hypothesis of stability of the underlying concept between the two assessments was more likely to be fulfilled. This study was also able to relate the GP5 ratings more closely to specific treatment-related events to document the construct validity of the GP5. These results corroborate previous findings showing strong associations between high bother as defined based on the GP5, with early treatment discontinuation, in patients undergoing treatment for multiple myeloma [25]. Higher GP5 ratings were observed in the assessments preceding treatment discontinuation, and to a lower extent for hospitalization. This less clear association with hospitalization may be explained by the fact that hospitalization could occur for any reason, regardless of symptomatic side-effects. Importantly, findings suggested that the GP5 can capture the difference in the burden associated with emerging symptomatic AEs reported by patients.

The second objective of the analysis was to confirm the categorization of “high side-effect burden” using a GP5 response of 3 “quite a bit” or 4 “very much”. Analyses of this categorization, and the comparison of two alternative categorizations, which consisted of either considering only those with 4 “very much” bother (alternative categorization #1) or adding those that reported 2 “somewhat” bother (alternative categorization #2), were supportive of the primary categorization. Patients categorized as experiencing “high side-effect burden” were consistently shown to have significantly poorer physical functioning and QoL. However, the greatest difference between the two groups was observed when high side-effect burden was defined using the “primary categorization” (i.e., having a GP5 of 3 or 4). Additionally, the objective when considering “high side-effect burden” was not to identify isolated episodes of extreme severe bother but rather to capture an experience that could last several weeks (data not shown). The results showed that a GP5 score of 4 maintained over two consecutive weeks was a rare occurrence. On the contrary, the categorization using a response of 3 or 4 to the GP5 led to periods of high side-effect burden that lasted a few weeks (mainly between 2 and 6 weeks), which was aligned with this objective.

The demonstration of the benefit of novel cancer therapies in terms of tolerability requires defining specific endpoints for “comparative tolerability.” The best definition for such endpoints is still under consideration. Two specifications for a comparative tolerability endpoint have been recently suggested targeting slightly different concepts [26], namely: the proportion of patients with high side-effect burden and the proportion of time with high side-effect burden. Both endpoint specifications are underpinned by the categorization of “high side-effect burden,” which the current analyses showed could be achieved with the GP5 item. Therefore, these analyses provide critical evidence to support the usefulness of this comparative tolerability endpoint in cancer clinical trials.

Despite the robust psychometric evidence generated by this research, a few limitations must be acknowledged. Firstly, a key component of the definition of tolerability relates to treatment adherence (the definition states “the ability or desire of the patient to adhere to the dose or intensity of therapy”). Establishing this relationship is particularly challenging, especially in the context of a clinical trial. Adherence to treatment in a study setting is particularly contrived, as patients follow a very strict, and closely monitored clinical trial protocol, which makes it difficult to obtain a measure of a natural adherence. Additionally, side-effect bother may not be the only driver to deter patients from following their prescribed treatment in a clinical trial setting, especially in a serious context where survival is at stake. This association may be investigated further in settings where a more direct association between treatment adherence and tolerability can be expected, either due to a non-life-threatening context or a less controlled environment. Another limitation of this study was that possible cultural variations in the performance of the GP5 were not explored, which may be relevant in the context of a global study, with some cross-cultural differences reported in previous research [9]. While the analyses were performed at different timepoints, the impact of time under treatment on the association between side-effect bother and treatment discontinuation was not assessed, as the present known-group analysis was performed regardless of the time of discontinuation. Previous work suggested increasing association with time on treatment [25], but this finding will need to be further confirmed. Also, while GP5 completion rates were satisfactory at post-baseline assessments (between 75% and 90%), the completion rate at baseline was 57%. The Baseline GP5 assessment has a recall period of the previous week, before participants start the treatment, which is likely to be challenging to interpret for the patient responding to the item. Findings from a qualitative study showed that treatment-naïve patients showed lower understanding and certainty in their GP5 response, as compared to those receiving treatment [27]. This may be a limitation for any analysis that used baseline GP5 data. Finally, it will be important to further confirm the categorization of “high side-effect bother” using qualitative evidence from interviews with patients. A qualitative stream of research was conducted to address this question, in parallel to the quantitative research reported here, which will be communicated in a separate paper.

## Conclusion

The psychometric analyses produced adequate evidence to support the use of the GP5 to assess patient-reported tolerability in MTC clinical trials. The categorization of “high side-effect burden” using a GP5 response 3 or 4 is appropriate for defining the comparative tolerability endpoint in LIBRETTO-531.

## Electronic supplementary material

Below is the link to the electronic supplementary material.

Supplementary Material 1 Supplementary Table 1. Baseline Demographic and Clinical Characteristics of the Safety Population

Supplementary Material 2 Supplementary Table 2. Correlations between EORTC QLQ-C30 functioning scores and GP5 at each cycle

Supplementary Material 3 Supplementary Fig. 1. Distribution of the GP5 at the 4 assessments before hospitalization and first visit after hospitalization, in relation to the benchmark group

Supplementary Material 4 Supplementary Fig. 2. Comparison of GP5 ratings at baseline and Cycle 2 Day 1 according to change in symptomatic adverse event in the Safety Population. Cochrane-Mantel-Haenszel test: *p*<0.0001

Supplementary Material 5 Supplementary Fig. 3. Comparison of GP5 ratings at baseline and Cycle 5 Day 1 according to change in symptomatic adverse event in the Safety Population. Cochrane-Mantel-Haenszel test: *p*<0.0001

## Data Availability

Eli Lilly and Company provides access to all individual data collected during the trial, after anonymization, with the exception of pharmacokinetic, genomic, or genetic data. Data are available to request 6 months after the indication studied has been approved in the US and EU and after primary publication acceptance, whichever is later. No expiration date of data requests is currently set once data are made available. Access is provided after a proposal has been approved by an independent review committee identified for this purpose and after receipt of a signed data sharing agreement. Data and documents, including the study protocol, statistical analysis plan, clinical study report, and blank or annotated case report forms, will be provided in a secure data sharing environment. For details on submitting a request, see the instructions provided at www.vivli.org.

## Abbreviations

GP5: Functional Assessment of Cancer Therapy item GP5
AE: Adverse event
EORTC: European Organization for Research and Treatment of Cancer (Item Library)
EORTC QLQ-C30: European Organization for Research and Treatment of Cancer Quality of Life Questionnaire – core 30 items
EQ-5D-5L: EuroQol Group’s 5-Dimension, 5-Level Descriptive System
FDA: United States Food & Drug Administration
GHS: Global health status
HRQoL: Health-related quality of life
ICC: Intraclass correlation coefficient
MTC: Medullary thyroid cancer
PRO: Patient-reported outcome (measure)
CTCAE: Common Terminology Criteria for Adverse Events
