# Supplementary material for: Assessing tolerability with the Functional Assessment of Cancer Therapy item GP5: psychometric evidence from LIBRETTO-531, a phase 3 trial of selpercatinib in medullary thyroid cancer
Source: J Patient Rep Outcomes. 2024 Dec 19;8:149. doi: 10.1186/s41687-024-00823-8 (PMC11655800; doi:10.1186/s41687-024-00823-8)
Supplement: Supplementary file 1 — Supplementary Material 1 Supplementary Table 1. Baseline Demographic and Clinical Characteristics of the Safety Population [file 41687_2024_823_MOESM1_ESM.docx]

Supplementary Table 1. Baseline Demographic and Clinical Characteristics of the Safety Population

| **Characteristic** | **Selpercatinib**  **(N = 193)** | **Cabozantinib or Vandetanib**  **(N = 97)** |
| --- | --- | --- |
| Age, years |  |  |
| Median | 56.0 | 53.0 |
| Range | 12 – 79 | 18 – 84 |
| Age distribution — n (%) |  |  |
| <18 years | 1 (0.5) | 0 |
| ≥18 to <65 years | 143 (74.1) | 72 (74.2) |
| ≥65 years | 49 (25.4) | 25 (25.8) |
| Sex — no. (%) |  |  |
| Male | 115 (59.6) | 67 (69.1) |
| Female | 78 (40.4) | 30 (30.9) |
| Race — no. (%)* |  |  |
| White | 116 (70.7) | 52 (66.7) |
| Asian | 43 (26.2) | 24 (30.8) |
| Black | 5 (3.0) | 2 (2.6) |
| Other | 0 | 0 |
| Missing | 29 | 19 |
| Region of enrollment — no. (%)^†^ |  |  |
| Europe | 109 (56.5) | 55 (56.7) |
| East Asia | 33 (17.1) | 20 (20.6) |
| North America | 12 (6.2) | 5 (5.2) |
| Other | 39 (20.2) | 17 (17.5) |
| ECOG PS — no. (%)^‡^ |  |  |
| 0 | 122 (63.2) | 54 (55.7) |
| 1 | 70 (36.3) | 39 (40.2) |
| 2 | 0 | 3 (3.1) |
| Missing | 1 (0.5) | 1 (1.0) |
| *RET* mutation — no. (%)^§^ |  |  |
| M918T mutation | 121 (62.7) | 61 (62.9) |
| Other mutations | 71 (36.8) | 36 (37.1) |
| Missing | 1 (0.5) | 0 |

* Race was reported by the patients. Black included African American. Presented as a percentage of subjects with non-missing data: selpercatinib (n=164) and cabozantinib or vandetanib (n=78).

† For geographic region of enrollment, Europe included Belgium, Czech Republic, France, Germany, Greece, Italy, Netherlands, Poland, Spain, United Kingdom, and Russia. East Asia included China, Japan, South Korea, and Taiwan. North America included Canada and USA. Other regions included Australia, Israel, Brazil, and India.

‡ Eastern Cooperative Oncology Group performance score (ECOG PS) is presented as a percentage of subjects with non-missing data: selpercatinib (n=192) and cabozantinib or vandetanib (n=96).

§ *RET* mutation is presented as a percentage of subjects with non-missing data: selpercatinib (n=192) and cabozantinib or vandetanib (n=97).
