# Supplementary material for: Assessing tolerability with the Functional Assessment of Cancer Therapy item GP5: psychometric evidence from LIBRETTO-531, a phase 3 trial of selpercatinib in medullary thyroid cancer
Source: J Patient Rep Outcomes. 2024 Dec 19;8:149. doi: 10.1186/s41687-024-00823-8 (PMC11655800; doi:10.1186/s41687-024-00823-8)
Supplement: Supplementary file 2 — Supplementary Material 2 Supplementary Table 2. Correlations between EORTC QLQ-C30 functioning scores and GP5 at each cycle [file 41687_2024_823_MOESM2_ESM.docx]

Supplementary Table 2. Correlations between EORTC QLQ-C30 functioning scores and GP5 at each cycle

|  | **Spearman correlations between GP5 and** | | | | | |
| --- | --- | --- | --- | --- | --- | --- |
| **Cycle** | **QLQ-C30 Physical functioning score** | **QLQ-C30 Role functioning score** | **QLQ-C30 Social functioning score** | **QLQ-C30 Emotional functioning score** | **QLQ-C30 Cognitive functioning score** | **QLQ-C30 Global health status/QoL score** |
| Cycle 1 | -0.11 | -0.15 | -0.17 | -0.16 | -0.16 | -0.15 |
| Cycle 2 | -0.57 | -0.56 | -0.63 | -0.46 | -0.36 | -0.56 |
| Cycle 3 | -0.56 | -0.58 | -0.58 | -0.54 | -0.45 | -0.56 |
| Cycle 4 | -0.45 | -0.48 | -0.49 | -0.45 | -0.39 | -0.51 |
| Cycle 5 | -0.56 | -0.57 | -0.61 | -0.41 | -0.45 | -0.50 |
| Pooled cycles | -0.46 | -0.48 | -0.50 | -0.39 | -0.37 | -0.47 |
