# Supplementary figures and images for: Assessing tolerability with the Functional Assessment of Cancer Therapy item GP5: psychometric evidence from LIBRETTO-531, a phase 3 trial of selpercatinib in medullary thyroid cancer
Source: J Patient Rep Outcomes. 2024 Dec 19;8:149. doi: 10.1186/s41687-024-00823-8 (PMC11655800; doi:10.1186/s41687-024-00823-8)

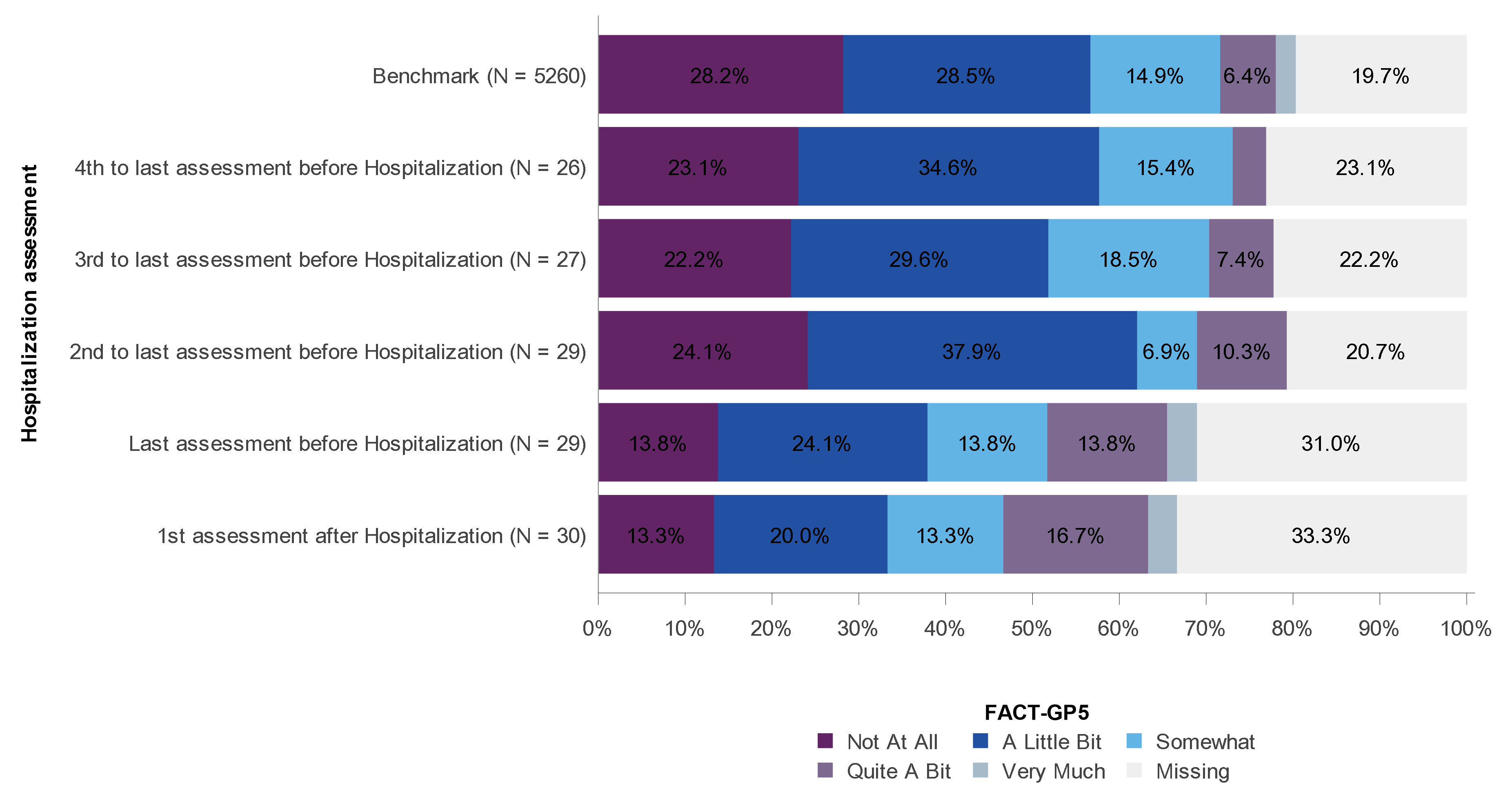

Supplement: Supplementary file 3 — Supplementary Material 3 Supplementary Fig. 1. Distribution of the GP5 at the 4 assessments before hospitalization and first visit after hospitalization, in relation to the benchmark group [file 41687_2024_823_MOESM3_ESM.png]

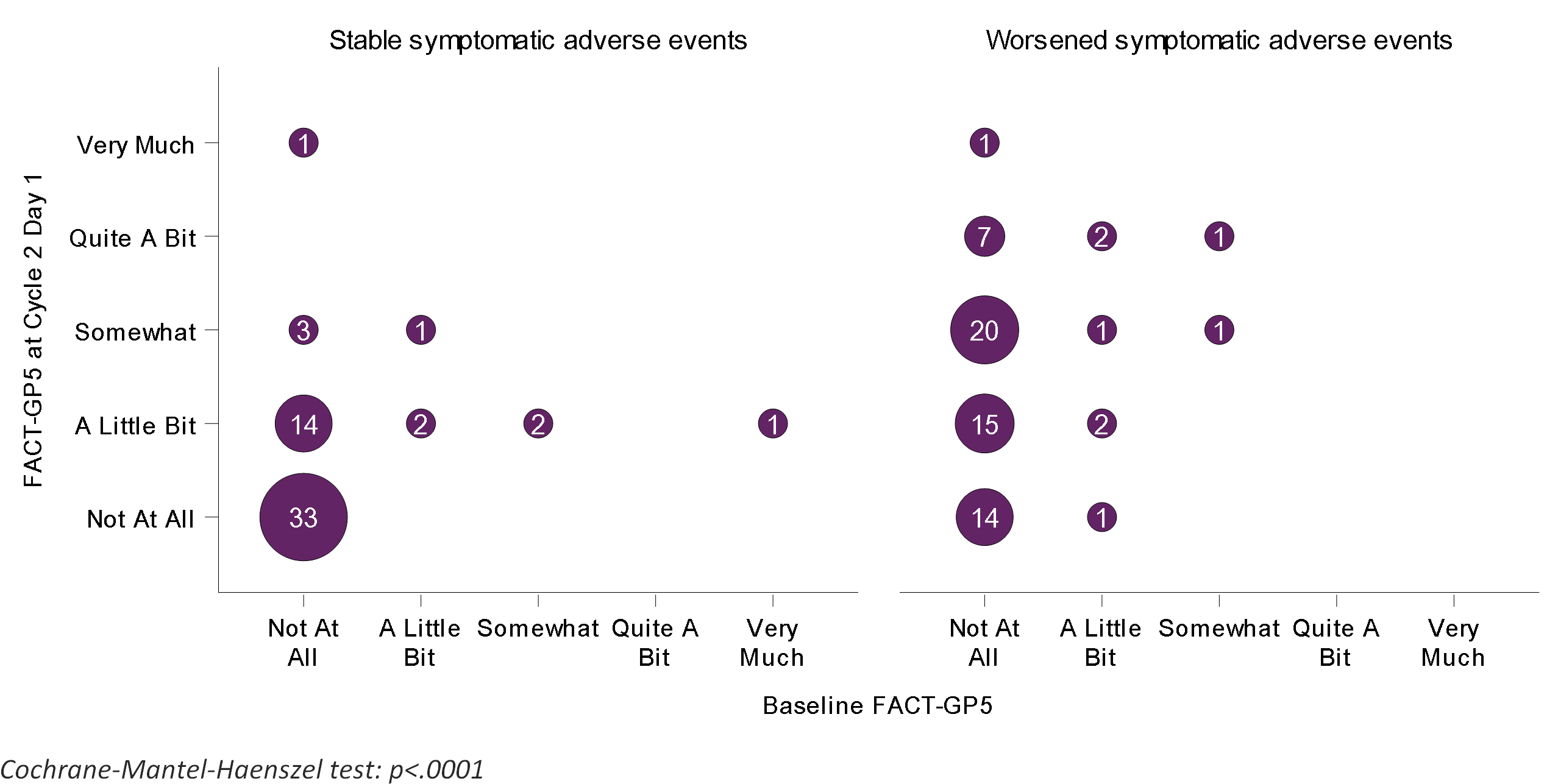

Supplement: Supplementary file 4 — Supplementary Material 4 Supplementary Fig. 2. Comparison of GP5 ratings at baseline and Cycle 2 Day 1 according to change in symptomatic adverse event in the Safety Population. Cochrane-Mantel-Haenszel test: p<0.0001 [file 41687_2024_823_MOESM4_ESM.png]

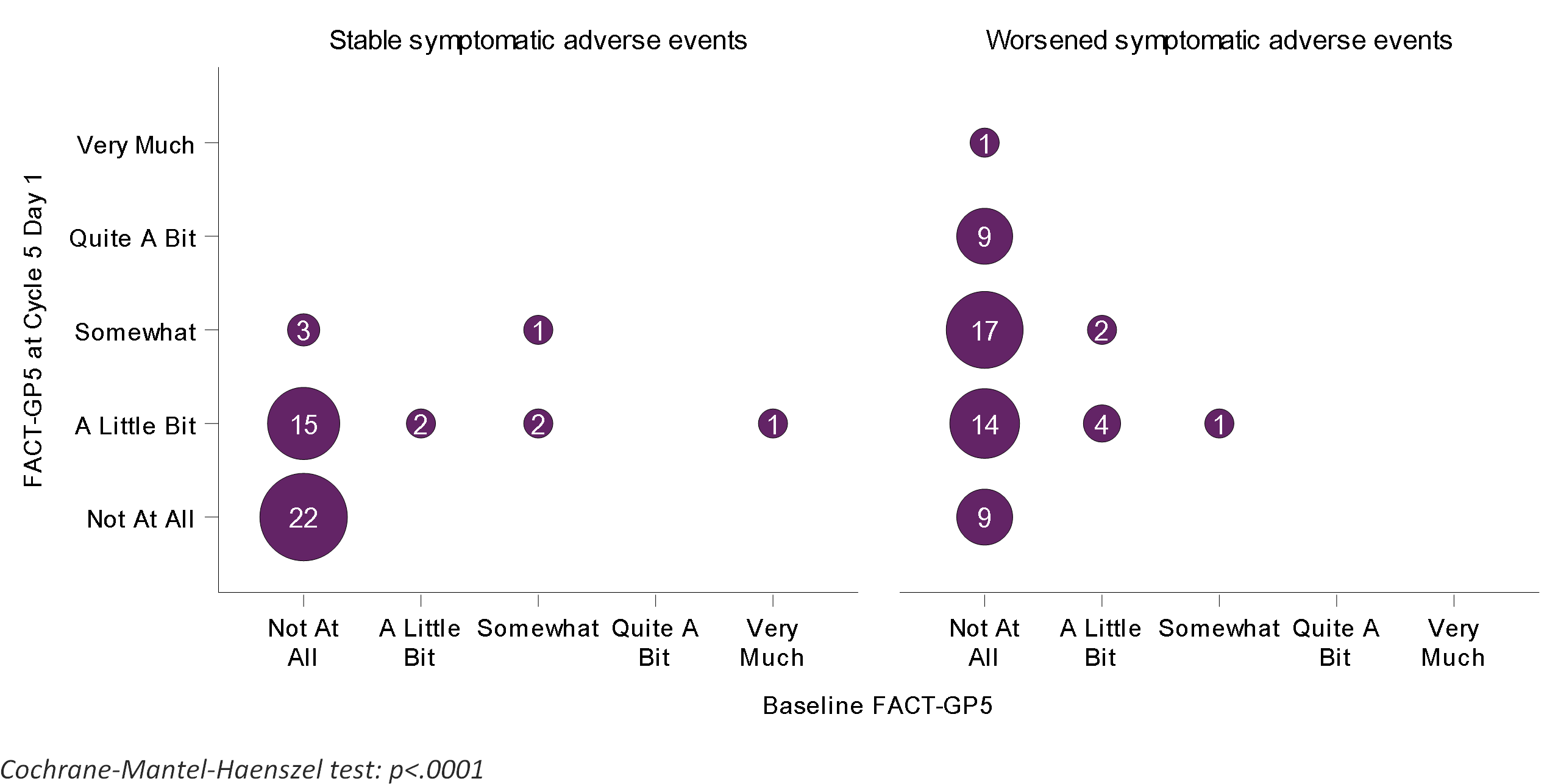

Supplement: Supplementary file 5 — Supplementary Material 5 Supplementary Fig. 3. Comparison of GP5 ratings at baseline and Cycle 5 Day 1 according to change in symptomatic adverse event in the Safety Population. Cochrane-Mantel-Haenszel test: p<0.0001 [file 41687_2024_823_MOESM5_ESM.png]
